# Intestinal stem cell aging signature reveals a reprogramming strategy to enhance regenerative potential

**DOI:** 10.1038/s41536-022-00226-7

**Published:** 2022-06-16

**Authors:** Christian M. Nefzger, Thierry Jardé, Akanksha Srivastava, Jan Schroeder, Fernando J. Rossello, Katja Horvay, Mirsada Prasko, Jacob M. Paynter, Joseph Chen, Chen-Fang Weng, Yu B. Y. Sun, Xiaodong Liu, Eva Chan, Nikita Deshpande, Xiaoli Chen, Y. Jinhua Li, Jahnvi Pflueger, Rebekah M. Engel, Anja S. Knaupp, Kirill Tsyganov, Susan K. Nilsson, Ryan Lister, Owen J. L. Rackham, Helen E. Abud, Jose M. Polo

**Affiliations:** 1 Department of Anatomy and Developmental Biology, Monash University, Clayton, VIC Australia; 2 Australian Regenerative Medicine Institute, Monash University, Clayton, VIC Australia; 3 Development and Stem Cells Program, Monash Biomedicine Discovery Institute, Monash University, Clayton, VIC Australia; 4 Institute for Molecular Bioscience, University of Queensland, St Lucia, QLD Australia; 5 Cancer Program, Monash Biomedicine Discovery Institute, Monash University, Clayton, VIC Australia; 6 Australian Research Council Centre of Excellence in Plant Energy Biology, School of Molecular Sciences, The University of Western Australia, Crawley, WA Australia; 7 Harry Perkins Institute of Medical Research, Nedlands, WA Australia; 8 Cabrini Monash University Department of Surgery, Cabrini Hospital, Malvern, VIC Australia; 9 Monash Bioinformatics Platform, Monash University, Clayton, VIC Australia; 10 Biomedical Manufacturing CSIRO, Clayton, VIC Australia; 11 Program in Cardiovascular and Metabolic Disorders, Duke-National University of Singapore Medical School, Singapore, Singapore; 12 Adelaide Centre for Epigenetics, The University of Adelaide, Adelaide, SA Australia; 13 The South Australian Immunogenomics Cancer Institute, The University of Adelaide, Adelaide, SA Australia

**Keywords:** Intestinal stem cells, Reprogramming, Ageing

## Abstract

The impact of aging on intestinal stem cells (ISCs) has not been fully elucidated. In this study, we identified widespread epigenetic and transcriptional alterations in old ISCs. Using a reprogramming algorithm, we identified a set of key transcription factors (*Egr1, Irf1, FosB*) that drives molecular and functional differences between old and young states. Overall, by dissecting the molecular signature of aged ISCs, our study identified transcription factors that enhance the regenerative capacity of ISCs.

## Introduction

Over the last two decades, it has been clearly demonstrated in many tissue systems that cellular identity is extremely plastic and can be widely manipulated^1^. Indeed, mature cell types can be reprogrammed back towards a pluripotent state by the forced expression of four transcription factors (TF)^2^. This paradigm demonstrates that cell differentiation and maturation are not unidirectional and, by extension, the ageing process as well. Several studies have shown that transient reactivation of *Oct4*, *Klf4*, *Sox2*, and *c-Myc* can restore or enhance stem cell potential^3,4^. Thus, in theory, similar reprogramming strategies could be used to reverse cellular traits associated with aging.

Epigenetic changes appear to be a key driver of the ageing process. Indeed, induced pluripotent stem cells derived from functionally compromised aged blood stem cells are able to generate young healthy chimeric animals with a normal hematopoietic system^5,6^, which supports the notion that the cellular ageing process is an epigenetic phenomenon that can be manipulated and reversed. Considering the control that TFs can have over the epigenome and determining cell identity, deciphering how the TF network is rewired during aging will provide a mechanistic understanding of age-related functional changes. This may reveal new ways to restore youthful regenerative potential. Since the impact of aging on intestinal cells has been recently described^7^, we utilized this model to study how age-related TF network changes underpin the aging phenotype.

The epithelium lining the intestine is the most highly cycling tissue in the body^8^. Epithelial renewal is driven by Lgr5-positive intestinal stem cells (ISCs) that reside at the bottom of the crypts^9^. Recent studies have shown that the decline in function with age is mediated in part by intrinsic alterations of Lgr5-positive ISCs^10,11^. This raises the question of whether the impact of aging can be directly reprogramed by manipulation of TF.

In this study, we confirm that aged ISCs have a reduced regenerative capacity in ex vivo organoid cultures. By performing comprehensive transcriptional analyses of ISCs, we uncovered the changes in the transcriptional network that underpin the age-reduced ISC regenerative response. A predictive reprogramming algorithm allowed us to identify three TFs capable of enhancing the regenerative potential of aged cells.

## Results

### Aging produces transcriptional and epigenomic changes in ISCs

**Fig. 1:**
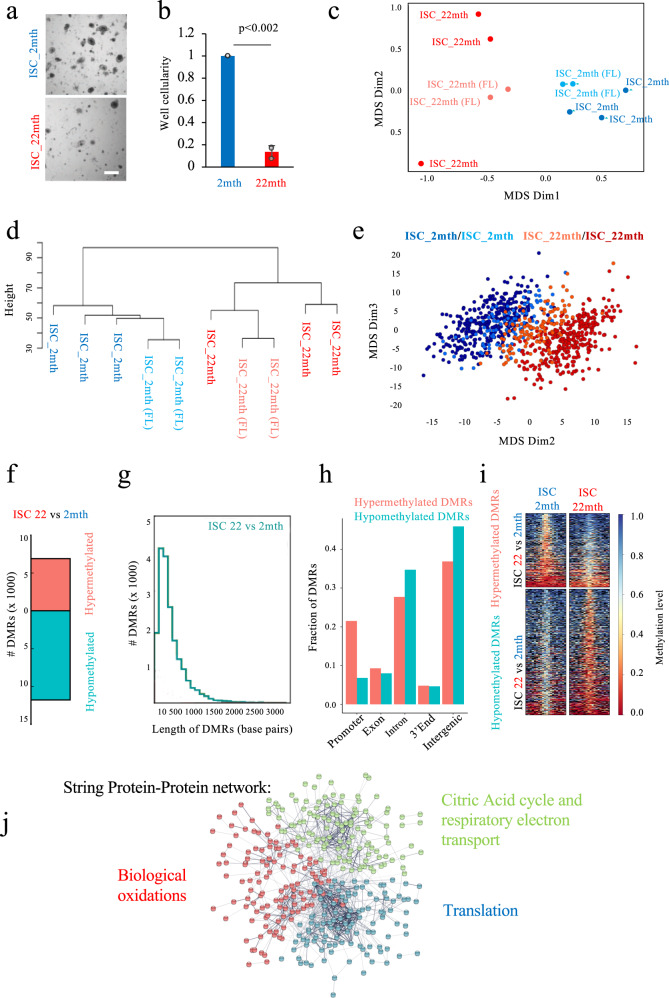
Molecular changes associated with ISC ageing. **a** Primary ISCs isolated from aged animals form less organoids in vitro compared to ISCs isolated from young animals (scale bar, 350 µm). **b** Quantification of well cellularity using the Presto Blue assay (Mean ± SEM, *n* = 3 experimental replicates, each data point per experimental replicate is the average of 1–2 biological samples isolated for each experiment, unpaired two-tailed Student’s *t* test). **c** Multidimensional scaling (MDS) analysis and (**d**) unsupervised clustering of RNAseq data for ISCs isolated from 2 and 22-month-old animals (*n* = 3 each using 3′-biased RNAseq approach with samples isolated from individual mice and *n* = 2 each from a pool of 5 animals using a full length [FL] transcript RNA-sequencing approach). **e** MDS analysis for single-cell sequencing data of ISCs coming from 2 and 22-month-old animals (*n* = 2, biological replicates). **f** Number of differentially methylated regions (DMRs) for pairwise comparison between 2 and 22-month-old ISCs and (**g**) the average length of DMRs for this pairwise comparison. **h** Genomic context and (**i**) heap map representation of hyper- and hypomethylated DMRs identified in 22-month-old ISCs compared to 2-month-old ISCs. Each horizontal heatmap slice represents 100 base pairs (**c**–**f**, *n* = 1, this *n* is derived from a pool of 5 animals). **j** String Protein/Protein interaction map of genes that are differentially expressed between young and old ISCs (*n* = 2, biological replicates coming from a pool of 5 animals for each *n*) with key gene ontology categories indicated.

In order to dissect age-related differences in stem cell function, we used the SM6 ISC isolation strategy^12^ that relies on the use of 6 cell surface markers (SM6), including CD24, EPHB2, CD166, and EPCAM, to discriminate between stem cells and their progenies in wildtype tissue. This approach enables the isolation of ISCs at purities equivalent to use of Lgr5-GFP reporter strain^12^. Single-cell transcriptional profiling of ISCs isolated from young and old WT mice confirmed the ability of the SM6 strategy to purify cells expressing key ISC markers including *Lgr5, Ascl2*, and *Sox9* irrespective of age (Supplementary Fig. 1a–d), with a percentage of *Muc2*+ lineage primed cells (Supplementary Fig. 1e) that is considerably lower than in single-cell studies of Lgr5-GFPhigh cells^13^. We assessed the functional capacity of purified ISCs to generate organoids in vitro. In agreement with previous studies^14,15^, ISCs isolated from old mice generated considerably less organoids than ISCs derived from young animals (Fig. 1a, b, Supplementary Fig. 2a). In addition, the size of organoids derived from old ISCs was 54% lower than their young counterparts (Supplementary Fig. 2b). These data demonstrate that the regenerative capacity of ISCs as well as the cellular proliferative potential is severely compromised in aging.

We then compared the expression profiles of young and old ISCs isolated from individual mice utilizing both the Lgr5-GFP reporter strain and the SM6 strategy. RNAseq analyses showed that both ISC isolation models share a common trajectory away from the young state with age (as per multidimensional reduction and correlation analyses; Supplementary Fig. 3a, b). To determine differentially expressed genes (DEGs) between young and aged WT ISCs at high sensitivity, we analysed the molecular changes in isolated ISCs in two pools of 5 animals for each age group (i.e., 10 animals per age group in total). This allowed us to procure cells in sufficient numbers to perform molecular assays in a paired fashion (RNAseq and DNA methylation) and to reduce the effects of individual inter-animal differences to reveal genuine and robust age-related differences. For RNAseq, we used a library preparation system that allows the detection of whole transcripts (not merely the 3-prime-end like for the data set displayed in Supplementary Fig. 3a, b). While both approaches identified the same ageing signature (Fig. 1c, d), the use of whole transcript libraries allowed us to detect subtle transcriptional differences associated with lowly expressed genes. ISCs have pronounced age-related changes, with 447 genes (FDR < 0.05) differentially expressed between young and old ISCs (Supplementary Table 1). Our scRNAseq data confirmed that aging induces a transcriptional shift away from the young ISC state (Fig. 1e). While dimension 1 of the MDS analysis primarily separated Muc2 negative cells according to differences in cell cycle (Supplementary Fig. 3c, d), dimension 2 separated cells according to age (Fig. 1e, Supplementary Fig. 3c), demonstrating that age-induced transcriptional changes are present even at the single-cell level.

To assess whether the ISC transcriptional changes could be explained by changes in the epigenome, we measured genome-wide DNA-methylation status (DNA-methyl-seq). ISCs acquired thousands of age-related differentially methylated regions (DMRs), specifically gaining DNA methylation at ~7000 loci and loss of DNA methylation at ~13,000 loci compared to their young counterparts (Fig. 1f–i). These data indicate that age-related changes in DNA methylation in ISCs are nearly an order of magnitude higher than the reported methylation changes that occur during differentiation of ISCs into Paneth cells in a young context^16^.

We then performed protein–protein interaction network analysis to determine whether the DEGs with aging have known interaction relationships and are enriched for coordinated biological processes. Our analysis showed a highly interconnected network associated with gene ontology categories related to energy metabolism and translation (Fig. 1j).

### Functional enhancement of ISCs by TF-mediated reprogramming

**Fig. 2:**
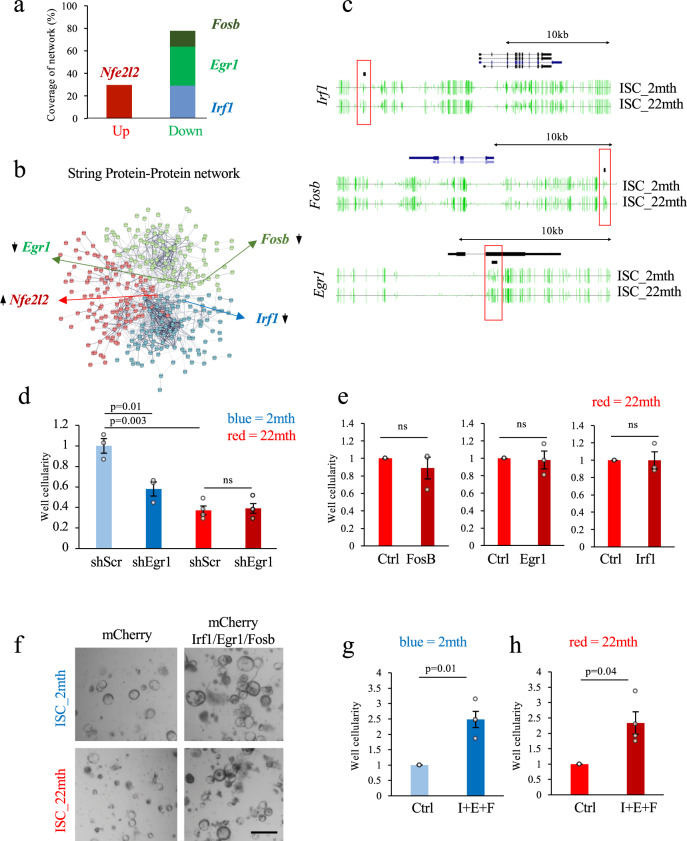
TF modulation boosts regenerative potential. **a** Visualization of the TFs coverage of the age specific ISC transcriptional network. **b** String Protein/Protein interaction map of genes that are differentially expressed between young and old ISCs with the location of Mogrify predicted TFs indicated; arrows indicated whether these TFs are significantly up- or downregulated in aged ISC. **c** UCSC DNA-methylation tracks (in green) for key TFs that are downregulated in aged ISCs. DMRs between 2 and 22-month-old ISC samples are indicated by black bars and associated regions are marked by a red frame (*n* = 1, this *n* is derived from a pool of 5 animals). **d** Organoid formation potential of secondary ISCs isolated from cultures that have been transduced with a scrambled shRNA control construct or shRNAs against *Egr1* (Mean ± SEM, 3–4 biological replicates for all experimental conditions, unpaired two-tailed Student’s *t* test). **e** Organoid formation potential of ISCs isolated from cultures that have been transduced with a mCherry control construct or constructs overexpressing *FosB, Egr1*, and *Irf1* (Mean ± SEM, 3 biological replicates for all experimental conditions, paired two-tailed Student’s *t* test). **f** Representative pictures of 2 and 22-month-old organoid cultures (scale bar, 200 µm) derived from ISCs transduced with mCherry control vector or a construct overexpressing *Irf1, Egr1*, and FosB (I + E + F). **g**, **h** Organoid formation potential of secondary ISCs isolated from organoid cultures from (**g**) 2 month and (**h**) 22-month-old mice that have been transduced with a mCherry control construct or a multicistronic construct co-overexpressing *Irf1, Egr1*, and *FosB* (I + E + F) (Mean ± SEM, 4 biological replicates for all experimental conditions, each data point per biological replicate is the average of two isolation experiments performed for the same transduced organoid cultures 2 weeks apart, paired two-tailed Student’s *t* test).

We next utilized our recently developed reprogramming algorithm (Mogrify) that predicts the TFs required for direct cell fate conversion^17^ to identify the key TFs driving aging in ISCs. Conceptually, the algorithm ranks the TFs within the DEGs between two cell states according to their influence over the regulatory network to identify the factors that enable direct reprogramming approaches. Using Mogrify, we identified a set of TF, *Nfe2l2*, *Irf1, Fosb*, and *Egr1* at the core of the transition from the young to aged ISC state (Fig. 2a, b). *Nfe2l2* was upregulated during aging, with a coverage of ~30% of the age specific transcriptome (Fig. 2a, b). Of note, *Nfe2l2* is associated with a protective response against oxidative stress^18^, indicating that the upregulation of this factor is likely an adaptive response to changes in cellular metabolism rather than a driver of these alterations. The remaining three predicted TFs *Irf1, Fosb*, and *Egr1* were downregulated during aging and cumulatively control potentially ~80% of the transcriptional network differences between young and aged ISCs (Fig. 2a, b). *Irf1* is a known mediator of interferon signaling^19^. *Fosb* is an early response gene, and, as part of the heterodimeric activating protein-1 transcription factor, is implicated in controlling proliferation, differentiation, and transformation^20^. Finally, *Egr1* orchestrates the expression of growth factors/receptors, matrix proteins and regulators of cell growth, controls differentiation, apoptosis and stress responses and has been implicated in modulating would healing and tissue regeneration in the liver, lung and skin^21,22^. While we identified 447 genes to be differentially expressed between old and young ISCs (Supplementary Table 1), the majority of these genes had no detectable changes in DNA-methylation status within or close to the gene body (Supplementary Fig. 4a). Conversely, *Irf1, Fosb* and *Egr1* in aged ISCs clearly possessed DNA hypermethylation associated with their gene bodies (Fig. 2c, Supplementary Fig. 4a). Using immunohistochemistry, we assessed the expression of these factors in situ. FOSB expression was observed at very low levels in both young and old crypts (Supplementary Fig. 4b). In contrast, we detected a stronger expression profile of IRF1 in young vs. old intestinal crypts (Supplementary Fig. 4c). We could not detect EGR1 expression under homeostatic conditions (Supplementary Fig. 4d). However, since *Egr1* is an early stress response gene, we reasoned that its expression might be elicited by the stress of tissue dissociation required to obtain samples for molecular profiling. Therefore, we assessed EGR1 expression in situ in intestinal tissue following injury induced by exposure to the antiproliferative drug 5-fluorouracil. EGR1 was detected 12 h after administration of 5-fluorouracil in young crypt sections, a response that was decreased in old crypts (Supplementary Fig. 4d).

We then hypothesized that manipulation of these 3 predicted TFs should blunt or augment the regenerative response of ISCs. To test this, we infected young and old organoid cultures with lentiviruses to induce knockdown or overexpression (Supplementary Fig. 5a). Knockdown of Egr1 only (but not of Irf1 or FosB on their own) decreased the well cellularity of secondary organoids generated from young ISCs (Fig. 2d, Supplementary Fig. 5b). Interestingly, Egr1 knockdown had no effect on organoid cultures established from old mice, indicating that the functional effects of age-induced Egr1 loss are not further enhanced by additional knockdown (Fig. 2d).

Forced expression of any of the three TFs individually in old organoids did not improve the ability of old ISCs to give rise to secondary organoids (Fig. 2e). As Mogrify predicted that all 3 TFs are required to achieve a network coverage sufficient to “reprogram” an aged cell into a young cell, we generated a multicistronic construct to co-express *Egr1, Irf1*, and *FobB* from one lentiviral backbone (Supplementary Fig. 6a), which elevated protein levels of all 3 TFs in transduced organoids (Supplementary Fig. 6b–d). Overexpression of the three TFs simultaneously in aged ISCs enhanced the regenerative potential, with 2–2.5 higher well cellularity (*p* = 0.01 and *p* = 0.04 respectively) compared to ISCs infected with a control vector (Fig. 2f–h). Interestingly, young ISCs also gained regenerative potential, indicating that those 3 TFs can augment ISC regenerative potential in both young and aged cells. In accordance with the cellularity measurements, overexpression of the three TF in both young and old stem cells increased organoid-forming potential (organoid count) and organoid size by 50% (*n* = 5, *p* < 0.01) (Supplementary Fig. 6e–h).

## Discussion

Decline in intrinsic stem cell function and/or supportive niche signals have been identified during aging in several organs. Classical studies in the mouse intestinal epithelium identified morphological changes occuring with age^23,24^ and more recently changes in cellular composition and stem and niche cell function have been identified^7^. Here, we define the epigenetic and transcriptional changes intrinsic to stem cells that underpin the age-related changes in the intestinal epithelium.

Our comprehensive molecular characterization of aged ISCs produced a clear picture of changes that accumulate with age allowing correlation of epigenetic changes and transcriptional signature. This enabled us to investigate a TF-based approach to augment regenerative potential and ameliorate decline in stem cell function.

Our reprogramming algorithm, Mogrify, identified three TFs (*Egr1, Irf1, Fosb*) that are downregulated in aged ISCs and are predicted to control up to 80% of the age specific ISC transcriptome network changes. Therefore, we tested the TFs predicted by Mogrify in combination to reach high levels of network coverage, as per the original publication. However, we cannot directly exclude that a combination of 2 factors might also positively affect organoid regenerative potential.

There is published evidence that links the function of all three TFs either to organismal lifespan or directly or indirectly to energy metabolism, which underpins a large proportion of the transcriptional changes between young and aged ISCs according to our gene ontology analyses (Fig. 1j). As such, it has been shown that *Egr1* overexpression extends lifespan in C. elegans^25^ and that it plays a role in lipid metabolism^26^. *Fosb* has been implicated with regulating systemic energy metabolism in the brain^27^. *Irf1*’s TF activity is controlled by its acetylation status which is mediated by NAD-dependent enzyme Sirt1^28^.

Genomic regions close to or within the gene bodies of *Egr1*, *Irf1*, and *Fosb* were hypermethylated with age, signifying that their downregulation (or inability to be upregulated during regeneration like in the case of *Egr1*) might have been orchestrated by changes to the epigenome acquired over time. To improve the age defective circuitry that is controlling ISC regeneration for therapeutic intervention, correcting age-related changes in the underlying epigenome for these key factors might offer a strategy to restore bona fide youthful function in future regenerative medicine applications. While our current data demonstrate that Irf1, Egr1, and FosB factor modulation can augment ISC regenerative potential in vitro, future in vivo work will be required to determine to what degree overexpression affects overall age-related transcriptional changes (e.g., Fig. 1c, e) and to what extent this restores a more youthful gene expression signature. Furthermore, studies on human organoid cultures will be required to confirm whether these factors, or another set of factors, have a similar effect on ISC regenerative capacity across species.

In summary, by using a reprogramming algorithm, we predicted and validated 3 TFs that control ISC regenerative capacity and are getting dialed down with age. As a model for our findings, we propose that age-induced epigenetic alterations of key genes shift the ISC TF network to a state of decreased regenerative potential. Collectively, we anticipate that our findings will open the door for future clinical applications in improving regeneration of the gastrointestinal tract.

## Methods

### Animal maintenance

Wild-type C57/Bl6 and Lgr5-EGFP-IRES-CreERT2 female mice were used. Animals were housed in specific pathogen-free animal house conditions at the animal facility (Monash Animal Research Platform) in strict accordance with good animal practice as defined by the National Health and Medical Research Council (Australia) Code of Practice for the Care and Use of Animals for Experimental Purposes as previously described. Experimental procedures were approved by the Monash Animal Research Platform Animal Ethics Committee. Animals were maintained under a 12/12 h light/dark cycle at a temperature of 20 °C with free access to food and water.

### 5-FU induced injury model

In order to induce injury in young and aged mice, a single dose of the chemotherapeutic drug 5-fluorouracil (150 mg/kg body weight, Sigma-Aldrich) that targets the proliferative cells of the intestinal epithelium was administered via an intra-peritoneal injection. Six mice were randomly selected from the aged and young animal groups and euthanised at 0 and 12 h post 5-FU administration. Following tissue isolation, the small intestine was flushed gently with cold phosphate-buffered saline (PBS), swiss-rolled and fixed overnight in 4% paraformaldehyde (Merck). The jejunum tissues were embedded in paraffin wax and sectioned at 4 μm onto Superfrost slides (Menzel-Glaser).

### Immunohistochemical analysis

Immunohistochemical staining was performed as previously described^29^. Briefly, slides were deparaffinised in xylene, rehydrated in graded alcohols and incubated in citrate buffer solution (pH = 6) for 10 min in a pressure cooker. Endogenous peroxidases were then blocked by incubating slides in 1% hydrogen peroxide for 5 min. After several washes with PBS, slides were blocked with CAS block (Life Technologies) for 1 h at room temperature before incubation overnight at 4 degrees with primary antibodies diluted in PBS containing 1% bovine serum albumin (anti-EGR1 antibody, 1/100, Cell Signaling, #4153; anti-FOSB antibody, 1/100, Cell Signaling, #2251; anti-IRF1 antibody, Abcam, 1/200, #ab186384). After 3 washes with PBS for 5 min each, slides were incubated with goat anti-rabbit or goat anti-mouse horseradish peroxidase conjugated secondary antibodies (1/200, Life Technologies) diluted in 1% BSA for 1 h at room temperature. After 3 washes with PBS for 5 min each, detection was visualized with DAB substrate chromogen (DAKO) for 1 min. Sections were then counterstained with hematoxylin for 2 min, dehydrated and mounted with DPX mounting medium (Sigma-Aldrich). The quantification of DAB signal intensity for EGR1, FOSB and IRF1 was performed using the software Fiji. Following color deconvolution, the average gray value within the crypt domains of DAB-stained pictures was calculated. The optical density per picture was determined as follow: OD = log (255/average gray value). The optical intensity was quantified in 30 crypt domains per animal.

### Isolation of intestinal cells

The isolated small intestine was flushed with PBS and opened longitudinally. The tissue was then scraped with a glass coverslip to remove villi, cut into 5-mm pieces and washed with PBS five times (20 inversions of the tube per wash). Following incubation for 30 min at 4 °C in 4 mM EDTA-PBS, intestinal crypts were released from small intestinal tissue fragments by mechanically pipetting with a 10 ml pipette in PBS and repeating this step twice. After centrifugation (430 × *g* for 5 min at 4 °C), the pellet was resuspended in PBS and strained using a 70-μm cell strainer (BD Biosciences). Following centrifugation (430 × *g* for 5 min at 4 °C), the collected crypts were incubated for 30 min at 4 °C in DMEM/F12–10% serum (Gibco), pelleted again by centrifugation (430 × *g* for 5 min at 4 °C) and then dissociated in TrypLE Express (Invitrogen) supplemented with 10 μM Rock inhibitor (Y-27632, Abcam) and 2.5 µg/ml DNAse 1 (Sigma-Aldrich) for 4 min at 37 °C. The dissociated cells were strained using a 70-μm cell strainer and were washed twice with PBS and collected by centrifugation at 4 °C at 430 × *g* for 5 min. Cellularized intestinal epithelial were labeled with antibodies and ISCs isolated by FACS (SM6 strategy, CD31^−^/CD45^−^/CD24^med^/CD166^+^/CD44^high^/GRP78^low^/EPHB2^high^/EPCAM^+^) as described previously in great detail^12,30^.

### Organoid culture initiated with purified ISCs

This was performed as described previously^31^. In brief, FACS-isolated ISCs were centrifuged (430 × *g* for 5 min at 4 °C) and the cell pellets resuspended in growth-factor reduced matrigel (1000 cells per microliter of Matrigel) (Corning, #356231). 5000 cells were seeded per well in a 96 well plate (4 technical replicates per condition). The culture medium consisted of basal crypt culture medium supplemented with 50 ng/ml EGF (Peprotech), 100 ng/ml NOGGIN (Peprotech), 1 µg/ml R-SPONDIN 1 (R&D Systems), 1 μM Jagged-1 (Genscript), 10 μM Y-27632 (Abcam), 100 ng/ml WNT-3a (Peprotech) and 2.5 μM CHIR (Stemgent). 100 µl of crypt culture medium was overlaid per well. ISCs were maintained in a 37 °C humidified atmosphere under 5% CO2. After 3 days, the culture medium was replaced by freshly made culture medium without Y-27632 and WNT-3a. After 4 days in culture, the number of cells per well was evaluated using the Presto Blue cell viability assay (Invitrogen). Organoids were incubated with Presto Blue solution for 20 min at 37 °C. The solution was then transferred into a new fluorescence-grade black 96 well plate and fluorescence measured using a plate reader (excitation: 540 nm; emission: 590 nm; BMG Labtech, Fluostar Optima). Organoids were then fixed with 5% formalin overnight and stained with DAPI for 30 min. Organoid number and area was quantified using the ImageXpress Pico automated cell imaging system (×4 magnification) and the CellReporterXpress image acquisition and analysis software (version 2.8.2.669). The inbuilt Cell Count module was used with the following settings: intensity—125, Minimum width—20, Maximum width—1000.

### Lentiviral transduction of organoid cultures

The multicistronic lentiviral constructs (e.g., Supplementary Fig. 6a) and shRNA carrying lentiviral constructs used in this study were purchased from Vectorbuilder. Knockdown experiments were performed with a pool of two shRNA lentiviruses against the same target gene. All constructs used in this study harbored a mCherry reporter fused to the open reading frame of the Neomycin resistance gene. Viral particles were produced as described previously^32^. Two days before transduction, established organoid cultures (directly established from crypt preparations) were re-plated and exposed to organoid media supplemented with 2.5uM CHIR (StemGent) and 10 mM Nicotinamide (Sigma-Aldrich) to obtain cystic, hyper-proliferative crypts. Treated organoid cultures were liberated from Matrigel and broken up into smaller pieces with a syringe using a 26-gauge needle. Organoid fragments were then dissociated into small cell clusters by incubation with TrypLE Express (Invitrogen) supplemented with 2.5 µg/ml DNAse I (Sigma-Aldrich) for 3 min, followed by quenching with 10 ml DMEM media containing 10% FBS. Cell clusters were spin inoculated (750 × *g*, for 1 h at room temperature) with high titer lentiviral preparations in organoid media supplemented with 2.5uM CHIR, 10 mM Nicotinamide and Polybrene (Millipore). Following spin inoculation and 3 h in a 37 °C incubator cell clusters were re-embedded in Matrigel in organoid media supplemented with 2.5 µM CHIR and, for the first 24 h, with Y-27632 (MedChem Express). After 5 days, CHIR was withdrawn and 100 μg/ml Geneticin (Sigma-Aldrich) added for positive selection of transduced cells.

### FACS isolation of ISCs from organoids

Organoid cultures were mechanically liberated from matrigel by frequent pipetting and then pelleted for 3 min at 450xg. After a washing step with PBS, followed by a second round of centrifugation, organoids were incubated with TrypLE Express (Invitrogen) supplemented with 2.5 µg/ml DNAse 1 (Sigma-Aldrich) and Y-27632 (MedChem Express) for 2.5 min and then cellularised, after addition of an equal volume of FBS, by gentle pipetting. The resulting cell pellets (3 min at 450 × *g*) were labeled, and ISCs positive for the mCherry reporter were isolated according to a modified version of our SM6 gating strategy^12^ for in vitro culture cells (for FACS enrichment strategy for mCherry+/CD24med/UEA1low/CD44high/EphB2high cells, see Supplementary Fig. 7 for gating strategy). Before submitting the sorted cells to the organoid formation assay (see section “Organoid culture initiated with purified ISCs”) we verified via FACS re-analysis that the sort cells had retained a high degree of viability (>80%).

### RNA sequencing and analysis

RNA was extracted with Qiagen’s RNeasy micro kit from 2–3 × 10^4^ FACS-isolated cells. For generation of sequencing libraries, 25 ng of RNA (RIN value >8) were submitted to SPIA amplification (NuGen). 2 biological pool replicates per condition were sequenced using the HiSeq 3000 sequencing platform (Illumina, San Diego, CA, USA). Of note young and old samples analyzed and presented in Fig. 1c, d using a full length (FL) transcript RNA-sequencing approach were derived from the cells of a pool of 5 animals per condition and replicate. Each library was single-end with a 50–100 nt read length. The targeted number of sequencing reads per sample was 30 million. Raw sequencing reads were assessed for overall quality using FASTQC (http://www.bioinformatics.babraham.ac.uk/projects/fastqc/). Sequencing specific adaptors and low quality reads (Phred score of 3 consecutive bases below 15, minimum read length of 36 nt) were filtered and hard trimmed using Trimmomatic [v 0.30]^33^. Where RNA-sequencing reads were 100 nt length, a crop to a length of 50 bp was performed (Trimmomatic [v 0.30]^33^.

For the RNA-sequencing data displayed in Supplementary Fig. 3a, b and Fig. 1c, d (excluding FL samples) a 3-prime biased approach was used to generate sequencing libraries^34^. An 8 bp sample index and a 10 bp unique molecular identifier (UMI) were added during initial poly(A) priming and pooled samples were amplified using a template switching oligonucleotide. The Illumina P5 (5′ AAT GAT ACG GCG ACC ACC GA 3′) and P7 (5′ CAA GCA GAA GAC GGC ATA CGA GAT 3′) sequences were added by PCR and Nextera transposase, respectively. The library was designed so that the forward read (R1) utilizes a custom primer (5′ GCC TGT CCG CGG AAG CAG TGG TAT CAA CGC AGA GTA C 3′) to sequence directly into the index and then the 10 bp UMI. The reverse read (R2) uses the standard R2 primer to sequence the cDNA in the sense direction for transcript identification. Sequencing was performed on the NextSeq550 (Illumina), using the V2 High-output kit (Illumina, #TG-160–2005) in accordance with the Illumina Protocol 15046563 v02, generating 2 reads per cluster composed of a 19 bp R1 and a 72 bp R2.

Sample reads were aligned to the mouse genome (GENCODE GRCm38 primary assembly) using STAR version 2.4.2a^35^. Transcript quantification was performed using featureCounts (exonic regions of GENCODE’s vM15 annotation version)^36^, and transcripts with more than 5 sequencing reads and 1 count per million of mapped reads in at least one sample were used for further analysis, previously library-size normalized using the TMM method^37^. Differential gene expression analysis was performed using limma voom with sample quality weights^38–40^. DEG were only determined for FL samples displayed in Fig. 1c, d.

Descriptive statistics and plots were analyzed and produced using limma^40^, EdgeR^41^, gplots version 3.0.1 (http://CRAN.R-project.org/package=gplots), and ggplot2 (https://cran.r-project.org/web/packages/ggplot2). Multidimensional scaling and principal component analysis was performed using limma’s plotMDS function and R on samples where gene read counts were scaled to the total number of sequencing reads per sample per million of mapped reads. Scaled gene read counts were log base 2 transformed (a moderation of 10 reads post scaling was used). Unsupervised hierarchical clustering (Pearson’s correlation) analyses were performed using limma^40^, bioDist (https://www.bioconductor.org/packages/release/bioc/html/bioDist.html) and hclust (https://stat.ethz.ch/R-manual/R-devel/library/stats/html/hclust.html) respectively. Comparative gene expression between 2 months ISCs and 22 months ISC and ISC NR was undertaken with edgeR (glmFit and glmLRT functions). Other statistical tests were performed as indicated in the figure legends.

The data displayed in Fig. 1c, d exhibited a technical batch effect, due to different times of sequencing and different library types (FL vs. 3′primed based). We overcame this by utilizing RUV^42^ normalization on negative control genes. We select genes that are both stable with respect to ageing and treatment conditions in the data. Specifically, with a maximum log fold change of 0.2, a minimum FDR of 0.5 and a minimum average expression (log CPM) of 5 in both contrasts. We proceed with 844 negative control genes to normalize the data using the RUVg method and k = 1.

### Single-cell RNA sequencing and analysis

Chromium controller (10× Genomics) cell isolation, encapsulation and library construction were performed as per the manufacturer’s instructions “Chromium Single-Cell 3′ Reagent Kit V2 User Guide”, 10X Genomics document number CG00052 Revision 3. A total of 12 cDNA amplification cycles were used. A total of 16 cycles of library amplification were used. Illumina sequencing was carried out using an Illumina NextSeq 500 using SBS V2 chemistry in high-output mode according to the recommendations outlined by 10X Genomics “Chromium Single-Cell 3′ Reagent Kit V2 User Guide”, 10X Genomics document number CG00052 Revision 3, with the exception that the second read was extended to 115b instead of 98b. Libraries were diluted according to the manufacturer’s instruction “NextSeq 500 System User Guide” Illumina document number 15046563 v02 and loaded at 1.8pM. Counts were produced using the 10X Genomics CellRanger pipeline v1.3.1, using the default settings. Data was analyzed and visualized using ASAP, a web-based platform for the analysis and interactive visualization of single-cell RNAseq data^43^.

### Mogrify predictions

Mogrify predictions between young and old ISCs were generated as described previously^17,44^ to identify key TFs that control the age specific transcriptome.

### Whole genome bisulfite sequencing by MethylC-Seq

Genomic DNA was isolated with the DNeasy Blood and Tissue Kit (QIAGEN, Cat#69504) according to manufacturer’s instructions. Libraries were prepared using NxSeq AmpFREE Low DNA Library kit (Lucigen, Cat#14000-1). Briefly, 200 ng sample genomic DNA with 0.5% (w/w) unmethylated lambda phage DNA (Thermo Fisher Scientific Cat#SD0011) was fragmented with a Covaris S220 sonicator to a mean length of 200 bp. Fragments were end-repaired, A-tailed, and ligated to methylated Illumina TruSeq adapters (BIOO Scientific, Cat#BIOO-511912) followed by bisulfite conversion using EZ DNA-methylation Direct kit (Zymo Research, Cat#D5024). Library fragments were then subjected to 5 cycles of PCR amplification with KAPA HiFi Uracil+ DNA polymerase (KAPA Biosystems, Cat#KP-KK2801). Single-end 100 bp sequencing was performed on a HiSeq 1500.

### DNA-methylation analysis

Single-end reads were trimmed using Trimmomatic-V0.32, to remove adaptor contamination and poor quality reads^33^. Trimmed reads were then aligned to reference mouse genome (mm10) using BSSeeker2 with default parameters^45^. Reads mapping to multiple locations were removed using sambamba-v0.5.9^46^. Thereafter, bs_seeker2-call_methylation.py module was used to call methylation levels. DMR identification was done using HOME version 0.4^47^. Briefly, HOME-pairwise module was used with *--delta* 0.3 and *--minc* 5 and rest parameters were kept as default for DMR calling. Gene ontology analysis was done using GREAT for hyper and hypomethylated DMRs^48^. Genome context of the DMRs was determined using Goldmine^49^. The heat maps for hyper and hypomethylated DMRs were generated using deepTools^50^. First, we used *computeMatrix* module of deepTools to compute the matrix from the ∆*mCG* in DMRs. The matrix was generated for 1 kb upstream and downstream of DMRs center. Thereafter, *plotHeatmap* module of deepTools was used to plot the heatmap.

### Western blot analysis

Organoid samples were pelleted at 450 × *g* for 1 min, supernatant was removed, and samples were washed twice with PBS followed by repeated pelleting. Samples were lysed with SDS-sample buffer (containing 10% (v/v) glycerol stock, 2% (v/v) SDS, 1.5% (wt/v) DTT, 0.005% (w/v) Bromophenol Blue in 62.5 mM Tris-HCl, pH 6.8) and protein content quantified using Pierce™ BCA Protein Assay kit (Pierce). Proteins samples were denatured at 95 °C for 10 min. SDS-PAGE runs were performed to separate proteins on a 4–15% Mini-Protean TGX gels (Bio-Rad) and transferred to a nitrocellulose membrane (Bio-Rad) for 1 h at 90 V. The membranes were blocked with 5% (w/v) milk or BSA in Tris-buffered saline with Tween-20 for 30 min at room temperature. Primary and secondary antibodies (Supplementary Table 2) were incubated overnight at 4 °C and 1 h at RT respectively; membranes were washed 3×, 5 min/wash after primary and secondary incubation using Tris-buffered saline with 0.1% Tween-20. Membrane was incubated with Clarity™ western ECL substrate (Bio-Rad) and imaged on a ChemiDoc™ MP Imaging System (Bio-Rad). Each blot or gel displayed derive from the same experiment and were processed in parallel.

### Reporting summary

Further information on research design is available in the Nature Research Reporting Summary linked to this article.

## Supplementary information

Supplementary Figures

REPORTING SUMMARY

## Data Availability

Sequencing data that support the findings of this study have been deposited in the Gene Expression Omnibus (GEO) database with the following accession code GSE198139.

## Code availability

Custom code used to generate results reported in the paper are available from the corresponding authors upon reasonable request.
