# Supplementary material for: Intestinal stem cell aging signature reveals a reprogramming strategy to enhance regenerative potential
Source: NPJ Regen Med. 2022 Jun 16;7:31. doi: 10.1038/s41536-022-00226-7 (PMC9203768; doi:10.1038/s41536-022-00226-7)
Supplement: Supplementary file 1 — Supplementary Figures [file 41536_2022_226_MOESM1_ESM.pdf]

Supplementary Figure 1: Related to Figure 1

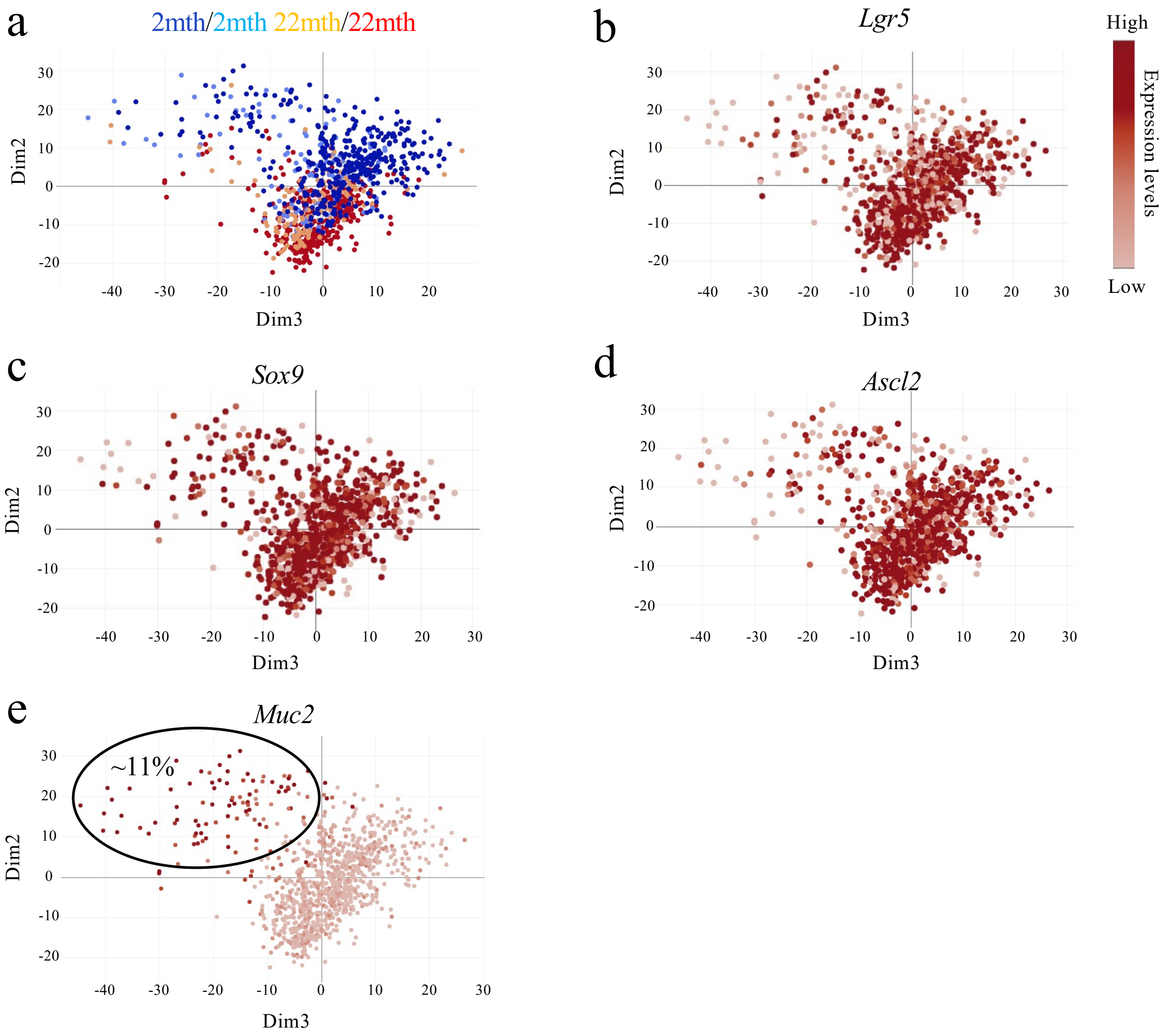

Supplementary Figure 1: Related to Figure 1. (a-e) MDS plots of single cell sequencing data set (a) indicating the location of individual replicates (biological replicates n=2 per age group) in the data set and the expression levels of (b-d) key ISC marker genes *Lgr5*, *Sox9* and *Ascl2* and (e) differentiation commitment marker *Muc2*.

Supplementary Figure 2: Related to Figure 1

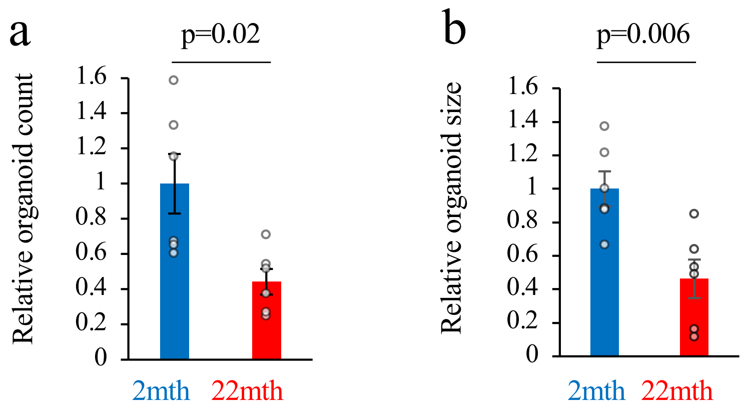

Supplementary Figure 2: Related to Figure 1. (a) Relative number and (b) relative size of primary organoids derived from FACS purified ISCs for the specified age groups (Mean ± SEM, n=6 biological replicates per age group, unpaired two-tailed Student's t test).

Supplementary Figure 3: Related to Figure 1

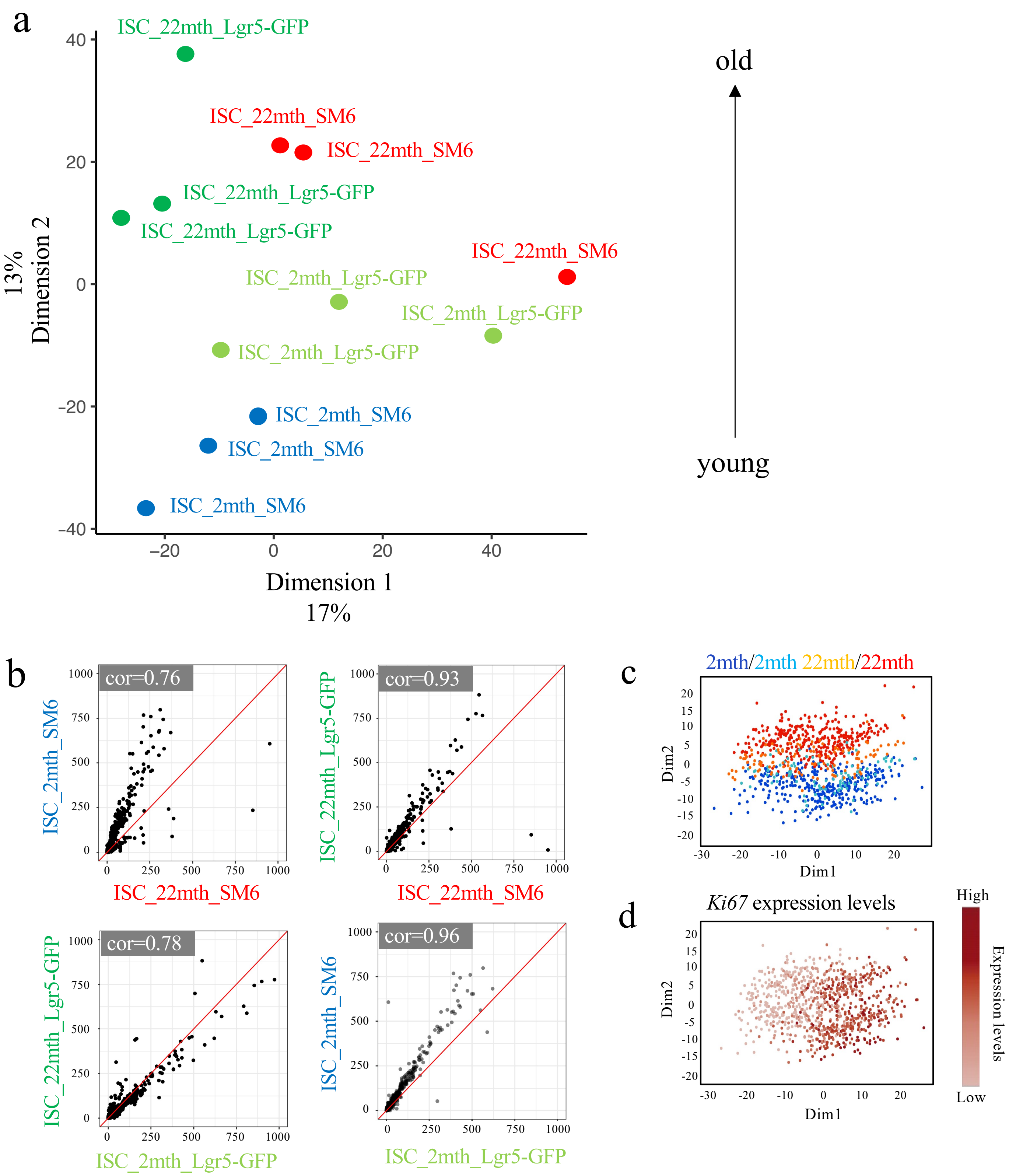

Supplementary Figure 3: Related to Figure 1. (a) Principal component analysis of for RNAseq data for ISCs isolated from individual 2mth and 22mth old animals. ISCs were either isolated from wild-type animals using the SM6 strategy (biological replicates, n=3 per age group) or from the Lgr5-GFP strain using the GFP reporter (biological replicates, n=3 per age group) . (b) Pairwise correlation analyses for the genes driving aging trajectory (biological replicates, n=3 per experimental group). (c.d) Additional MDS plots for single cell sequencing data set after depletion of *Muc2* positive cells indicating (c) location of the cells from the 2 biological replicates per age group and (d) expression levels of *Ki67*.

Supplementary Figure 4: Related to Figure 2

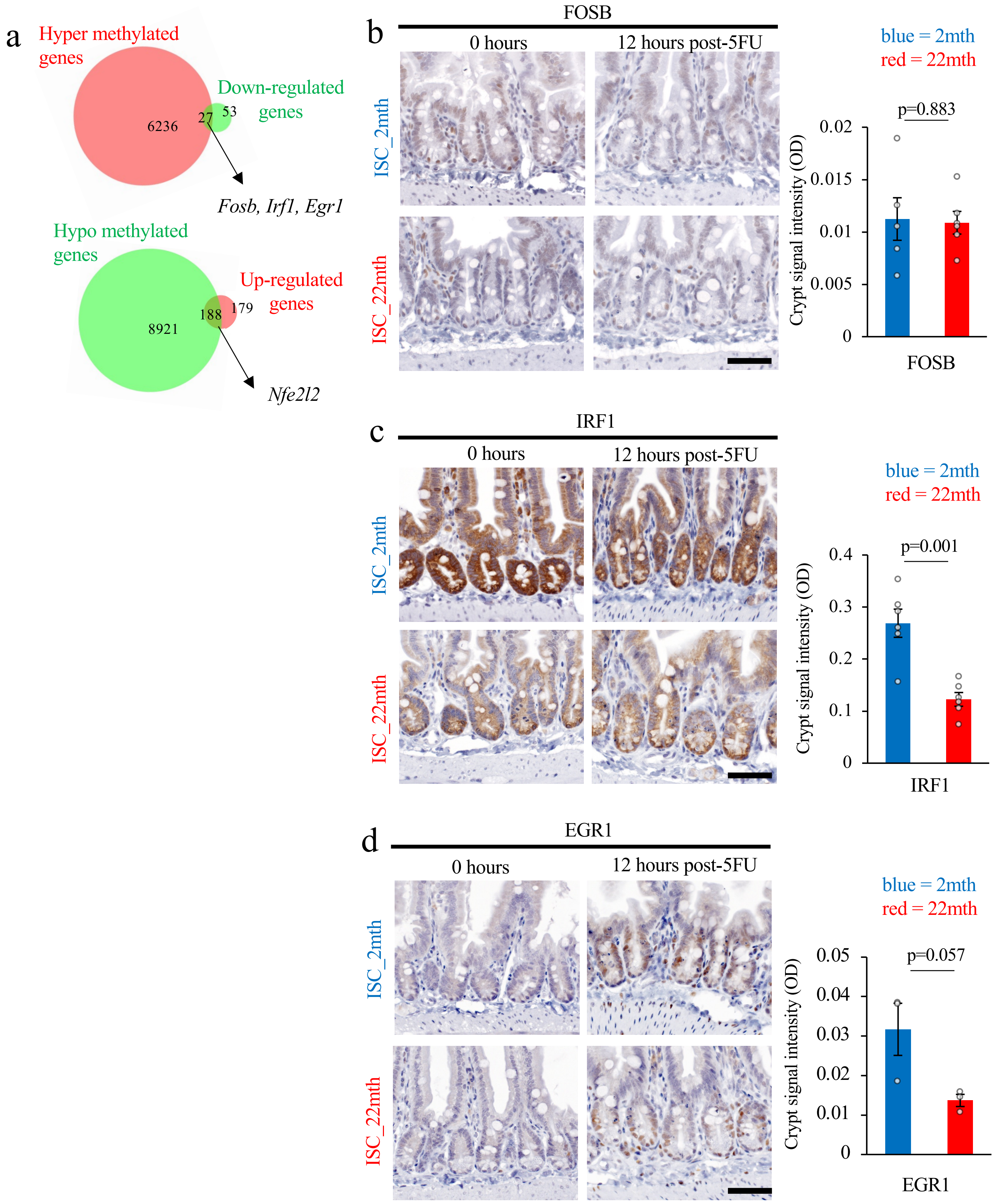

Supplementary Figure 4: Related to Figure 2. (a) Overlap between genes that gain or lose DNA methylation status in aged ISCs and genes that are down-regulated or up-regulated at the transcriptional level in aged ISCs respectively. (b-d) Representative pictures (scale bar, 20μm) and quantification of signal intensity of tissue sections labelled for (b) FOSB, (c) IRF1 and (d) EGR1 from 2mth and 22mth old animals under homeostatic conditions or 12 hours after treatment with chemotherapeutic agent 5-FU; for FOSB and IRF1 quantifications were performed under homeostatic conditions (Mean ± SEM, 5-6 biological replicates per group, unpaired two-tailed Student's t test) , for EGR1 quantification was performed 12 hours after treatment with 5-FU (Mean ± SEM, 3 biological replicates per group, unpaired two-tailed Student's t test).

Supplementary Figure 5: Related to Figure 2

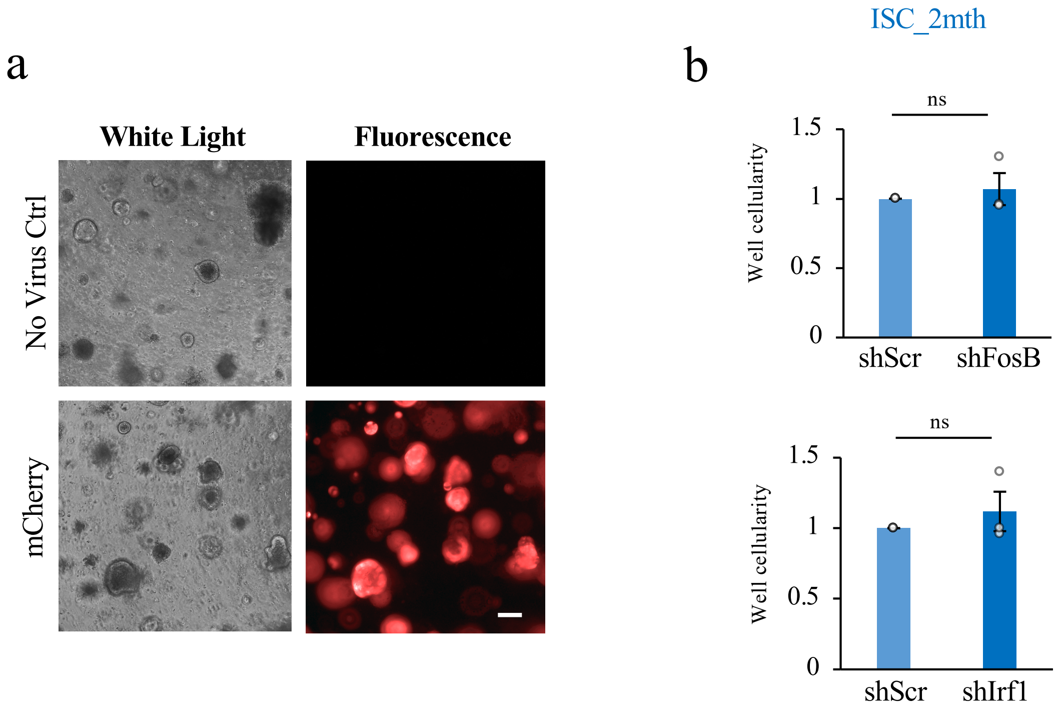

Supplementary Figure 5: Related to Figure 2. (a) Representative pictures of organoid cultures infected with or without a mCherry expressing lentiviral construct (scale bar, 200µm). (b) Organoid formation potential of secondary ISCs isolated from cultures that have been transduced with a mCherry control construct or constructs expressing shRNAs targeting *FosB or Irf1* (Mean ± SEM, 3 biological replicates for all experimental conditions, unpaired two-tailed Student's t test).

Supplementary Figure 6: Related to Figure 2

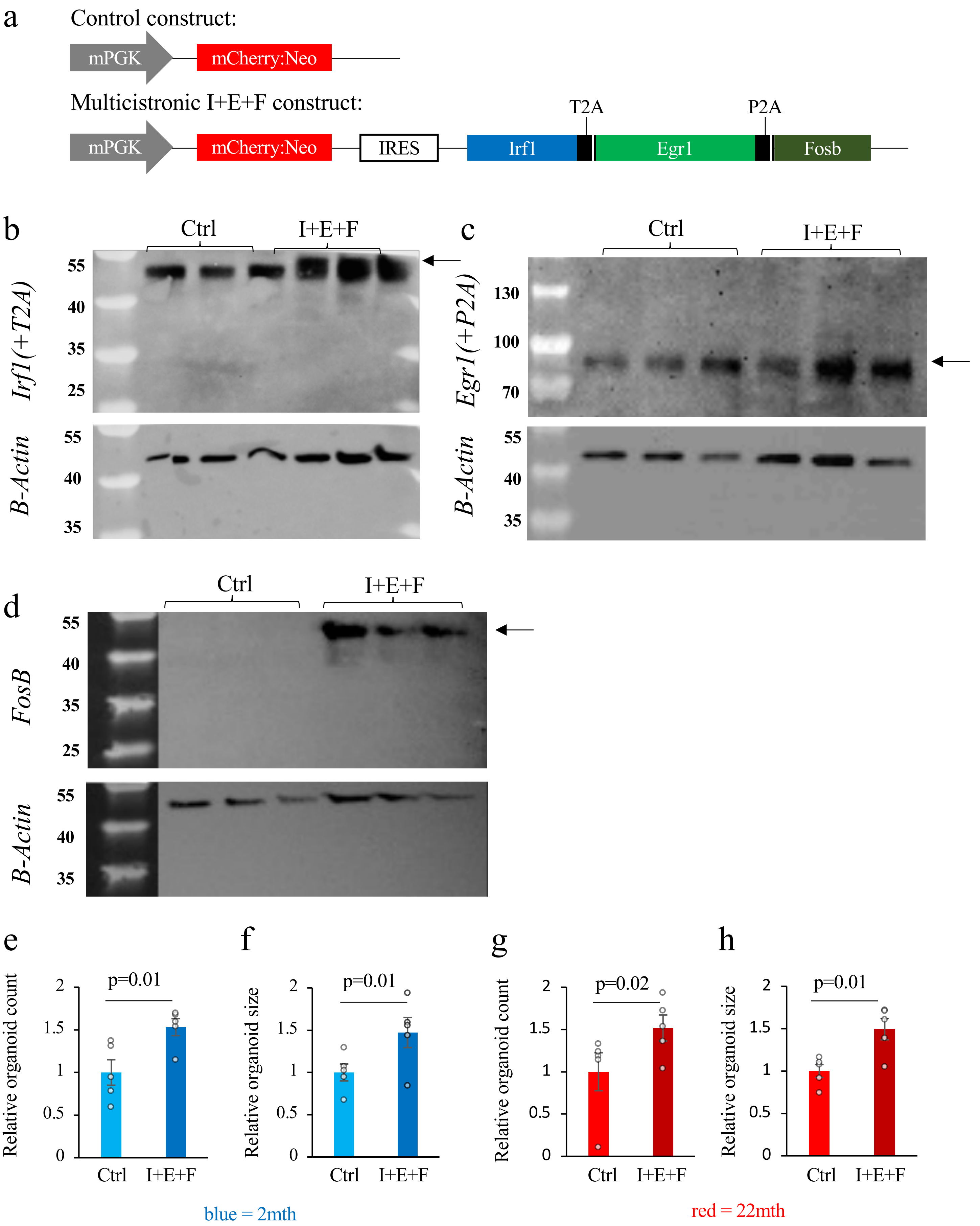

Supplementary Figure 6: Related to Figure 2. (a) Schematic of expression cassette (including control construct) used to overexpress Irf1, Egr1 and FosB in intestinal organoids via lentiviral transduction. (b-d) Chemiluminescent Western blot scans for (b) Irf1, (c) Egr1 and (d) FosB for protein extracts from aged organoids transduced with the control of multicistronic I+E+F contruct. Irf1 and Egr1, expressed via the multicistronic construct, have fusion tags (parts of T2A and P2A element) and as such are slightly larger than wild type proteins (n=3, biological replicates). For panel d, the protein ladder (lane 1), could not be clearly detected via chemiluminescent scanning, but clearly via colorimetric scanning, therefore the ladder sections for (d) are provided in the form of colorimetric scans. (e-f) Relative (e) number of organoids and (f) organoid size per condition (Mean ± SEM, 5 biological replicates per group, paired two-tailed Student's t-test) linked to the experimental conditions displayed in Figure 2f,g. (g-h) Relative (g) number of organoids and (h) organoid size per condition (Mean ± SEM, 5 biological replicates per group, paired two-tailed Student's t-test) linked to the experimental conditions displayed in Figure 2f,h.

Supplementary Figure 7: FACS gating strategy to enrich for ISCs from *in vitro* cultured organoids

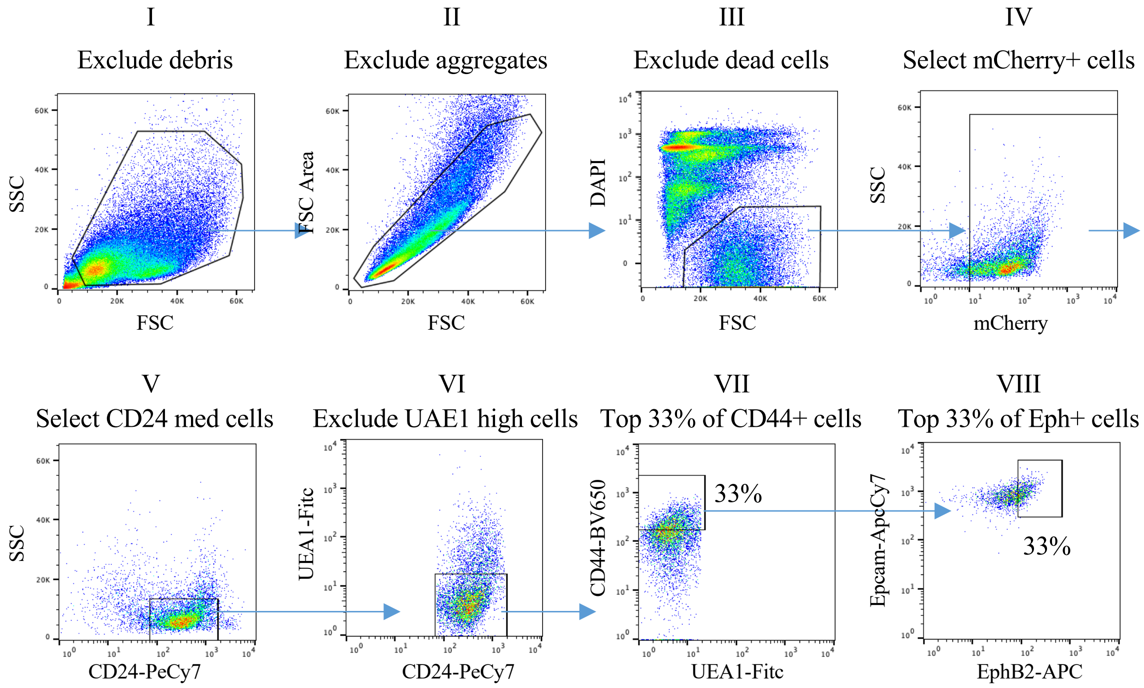

Supplementary Figure 7: FACS gating strategy to enrich for ISCs from *in vitro culture* organoids. Following exclusion of debris, cell aggregates, dead cells and mCherry negative cells (I-IV), CD24 med cells were selected (V) followed by a depletion step for UEA1+ Paneth cells (VI). Afterwards ISCs were enriched by gating in on the top ~33% of CD44 high cells (VII) followed by selecting the top 33% EphB2 high population from the Epcam+ cells (VIII). Please note that due to slight differences in labelling intensity gates need to be re-adjusted for each new sort sample.

Supplemental Table 1: Differentially expressed genes young vs aged ISCs

Gene Name	ISC_2mth	ISC_22mth	FDR
Ptgs1	0	6.050659122	0.014994821
P2rx7	0	5.60081682	0.004044313
Ddr2	0	5.317508622	0.02492109
Ccr2	0	5.116944008	0.027764553
H2-Eb1	0	4.426758948	0.028074029
Ceacam10	0	3.904168988	0.000821237
Gad2	0	3.544624116	0.017316415
Tenm4	0	3.456451847	0.000275923
Tspan18	0	3.131696561	0.018510439
Parp3	0	3.08089167	0.022181277
2210417A02Rik	0	2.929108181	2.74E-05
Dclk1	0	2.920495562	0.044204522
Gm17619	0	2.546582962	0.03936931
mt-Tc	0	2.485921678	0.044344278
H2-Aa	0	2.480751217	0.030908045
Cd74	0	2.43040482	2.35E-05
Fabp1	0	2.278093163	8.64E-06
Slfn4	0	2.190652574	0.036008007
Fmo1	0	2.180496327	0.017718898
Cpe	0	2.088850064	0.046692758
Anks6	0	2.041427199	0.024457385
Cnpy1	0	1.930330432	0.035422979
Cyp4a32	0	1.909554731	0.026451656
Cela1	0	1.874293849	0.030510978
Aim2	0	1.84665313	0.004334551
Gas7	0	1.824698043	0.000371699
Ly6e	0	1.775020058	6.90E-09
2010106E10Rik	0	1.737762644	0.04626163
Rpgrip1	0	1.725409579	0.017718898
Aldh1a1	0	1.708352415	0.004423948
Akr1c19	0	1.69250492	0.009206317
Gm10451	0	1.691985857	0.033593555

Slc7a9	0	1.685210496	0.007880404
Pla2g5	0	1.658776913	0.010029422
Glt1d1	0	1.646147548	0.035497641
Ddo	0	1.640246004	0.03705273
Syna	0	1.620730855	0.000500928
Ppic	0	1.586290664	0.000110881
Rgs7bp	0	1.559286916	0.008047997
Sulf2	0	1.5473504	0.028074029
mt-Tl1	0	1.522798396	2.35E-05
Mep1b	0	1.503249936	0.003856852
mt-Tm	0	1.479936432	0.04626163
Car4	0	1.458118746	0.000412721
Card6	0	1.45625124	2.35E-05
Rpl15	0	1.391313354	0.006333473
Sis	0	1.359598211	0.000223779
mt-Nd2	0	1.350303879	2.76E-05
Acot1	0	1.349287239	1.23E-05
Tmed6	0	1.340547895	0.033593555
Gm26529	0	1.32028079	0.030908045
Cyp2c65	0	1.279855146	0.000763377
Gsta4	0	1.273252813	0.008195088
Gm6086	0	1.268007547	0.028064966
Gp1bb	0	1.247204546	0.027624471
Chst2	0	1.245673988	0.009206317
Gm37310	0	1.228031727	0.027297607
Samd9l	0	1.198691072	0.027297607
AI427809	0	1.193038305	0.003715099
Reg1	0	1.184464365	0.008749016
Ace2	0	1.176400042	0.016120511
Phgr1	0	1.152785216	0.027624471
Cd55	0	1.151574897	0.00741607
Ablim1	0	1.145920914	0.011208801
Maf	0	1.137599322	0.038962099
Enpep	0	1.131473058	0.001544224

Slc1a3	0	1.129701434	0.000423929
mt-Nd6	0	1.112485818	5.01E-05
mt-Nd5	0	1.099886685	8.64E-06
Ifi47	0	1.096009444	0.001328939
Ugt2a3	0	1.080792809	0.042907876
Tst	0	1.073216308	0.01171753
Papss2	0	1.064583405	0.003945494
Rdh14	0	1.057977853	0.01982306
Clec2h	0	1.035243447	0.012151167
Sycn	0	1.028107799	0.013171118
Defa17	0	1.012681326	0.016455163
Cyp2e1	0	1.005540676	0.011312479
Rbp2	0	0.98244428	0.044344278
mt-Nd1	0	0.975072217	8.64E-06
Fabp2	0	0.973043874	0.002860964
Gm15284	0	0.969569435	0.012908883
Acadl	0	0.952673933	0.000821237
mt-Nd4	0	0.943082135	8.64E-06
Fmo4	0	0.931018865	0.03705273
Tmem177	0	0.928091863	0.044344278
Hist1h2bc	0	0.922982842	0.01422218
Cast	0	0.921497315	0.000110881
Rpa3	0	0.911862737	0.03936931
Stox2	0	0.901640233	0.03573349
A130010J15Rik	0	0.900384002	0.033593555
Ndufc1	0	0.900294764	0.004044313
Sec61b	0	0.897863922	0.031481217
Sult1b1	0	0.883690955	0.003042009
mt-Cytb	0	0.856421008	0.000368941
Gm13340	0	0.85327236	0.002588921
mt-Co1	0	0.849725494	0.000227776
Gm44250	0	0.8417305	0.004044313
Fam111a	0	0.840770099	0.007880404
Afap1	0	0.828120538	0.031430844

Nipsnap3b	0	0.825918743	0.014712583
Mospd1	0	0.819561227	0.022767341
Ccnc	0	0.805360421	0.035195113
Msantd4	0	0.80500754	0.010356742
Galnt12	0	0.803720649	0.007012607
Ifitm3	0	0.803609339	0.007990316
Ces2e	0	0.801784362	0.024534138
Oxnad1	0	0.795401298	0.044344278
Fahd1	0	0.79539819	0.00987359
Clic5	0	0.795196372	0.03936931
Defa24	0	0.792552632	0.033593555
Casp4	0	0.791351187	0.019764378
Itgam	0	0.790116502	0.031708824
Uqcrfs1	0	0.787749543	0.000368941
Rab4a	0	0.778785576	0.027639787
Ndufa1	0	0.778291446	0.033593555
Ces2g	0	0.772270978	0.001169923
Txndc17	0	0.767024175	0.000434255
Nampt	0	0.764039838	0.02492109
Rnase1	0	0.76063057	0.004044313
Gatc	0	0.75935161	0.003653421
Hmgcs2	0	0.755180914	0.01394245
Akr1c12	0	0.754891787	0.011849132
Ptcd2	0	0.751740862	0.018021988
Prr15	0	0.750240852	0.026602774
Gm24357	0	0.748733477	0.035422979
Cradd	0	0.743828182	0.045372544
Marcks	0	0.743448356	0.000105764
Ywhaq	0	0.742082549	0.006333473
Fdx1	0	0.740800135	0.038962099
Ebag9	0	0.739393963	0.011208801
Slc25a24	0	0.736596569	0.014068589
Skap2	0	0.736339351	0.037866868
Utp11l	0	0.731283199	0.033593555

Nipal1	0	0.728963614	0.042770258
Sec11c	0	0.725547358	0.006845141
Idh1	0	0.71836321	0.013231376
Cmpk1	0	0.716333482	0.002746706
Calm2	0	0.712021439	0.001983028
Ppig	0	0.710086231	0.000155406
Gclm	0	0.704997862	0.008749016
Maoa	0	0.702302993	0.010356742
Prss32	0	0.702093839	0.002025382
Slc6a4	0	0.702039446	0.009835879
Dld	0	0.695595735	0.000368941
Glrx3	0	0.694965611	0.042728076
Cd24a	0	0.69176298	2.35E-05
Snx6	0	0.690762489	0.033593555
Ndufa6	0	0.689710892	0.04456691
Pkp2	0	0.686001104	0.000763377
Sav1	0	0.684828651	0.04146396
mt-Rnr1	0	0.678159215	0.000139789
Coro2a	0	0.669144032	0.001085044
Lypd8	0	0.66898865	0.0042246
mt-Rnr2	0	0.667455823	0.000144993
Jagn1	0	0.66728908	0.009263981
Laptm4b	0	0.667164995	0.047693037
42248	0	0.666879745	0.009368624
Gstm3	0	0.665625768	0.028918015
Smim15	0	0.6629462	0.027265512
Inpp5f	0	0.660543274	0.028139897
Ssb	0	0.652920341	0.001846714
Uprt	0	0.652858209	0.037285233
Tmed5	0	0.652036	0.007386026
Nipal2	0	0.647073579	0.042770258
Ccl28	0	0.646844654	0.009368624
Apobec3	0	0.646757217	0.006407305
Bche	0	0.644370631	0.001085044

Sgf29	0	0.642306059	0.042770258
Phyh	0	0.641557207	0.005809024
Mrpl30	0	0.641064183	0.010356742
Mrpl36	0	0.640572153	0.011648426
Nxt2	0	0.638039409	0.008749016
Cat	0	0.637310274	5.83E-05
Prr13	0	0.633513706	0.017718898
Bpnt1	0	0.632646113	0.04626163
Ndufaf4	0	0.632306648	0.049946816
Ctsh	0	0.631091331	0.012856171
Chordc1	0	0.627723315	0.014712583
Esd	0	0.624413434	0.012759988
Tmem30a	0	0.617511326	0.039916856
Snx2	0	0.613557959	0.028413444
Casp1	0	0.613447477	0.004876655
Perp	0	0.612382758	0.017521409
Eny2	0	0.609808387	0.009206317
Mylk	0	0.609305582	0.013089951
Tlr3	0	0.606829705	0.017316415
Vti1a	0	0.605793218	0.031496527
Ola1	0	0.604882318	0.003187153
5330417C22Rik	0	0.604292187	0.008047997
Rnaseh2b	0	0.603772562	0.029400943
Ptges3	0	0.60183276	0.027004002
Mdh1	0	0.600664696	0.002700826
Rbbp8	0	0.598591396	0.034786995
Psma2	0	0.595375453	0.010356742
Slc31a1	0	0.595373395	0.0042246
Nifk	0	0.595274982	0.033593555
Xrcc5	0	0.59452193	0.037927486
Slc30a4	0	0.593901708	0.025954897
9130409J20Rik	0	0.591680577	0.040626833
Npm1	0	0.591129432	0.000624173
Slc39a8	0	0.589880068	0.03398164

Sh3bgrl	0	0.588018015	0.006333473
Ube2d2a	0	0.586932792	0.046692758
Atf1	0	0.586308728	0.009263981
Scarb2	0	0.586187538	0.002860964
Rfk	0	0.585545804	0.004876655
4931406C07Rik	0	0.583630529	0.005809024
Lap3	0	0.581699264	0.044344278
Chpt1	0	0.580211917	0.014068589
Nop58	0	0.579382191	0.000624173
Otc	0	0.579195762	0.001983028
Gsr	0	0.577809702	0.009150885
Cacybp	0	0.575688739	0.042122445
Adh1	0	0.575457705	0.039537516
Tmem45b	0	0.568680589	0.033991007
Sgpp1	0	0.563784154	0.0042246
Smco4	0	0.562863903	0.040245425
Mapk1ip1l	0	0.562434122	0.00236292
Mgst1	0	0.562021855	0.042907876
Pls1	0	0.556919301	0.004044313
Krr1	0	0.556707018	0.033593555
Zfp422	0	0.555534267	0.030523415
Rrm2	0	0.55458251	0.034710344
Guf1	0	0.553455343	0.033593555
Ndufa8	0	0.552479984	0.035953368
Tmed9	0	0.552431628	0.006629214
Fbxo9	0	0.552058804	0.046767389
Cops5	0	0.549728191	0.027297607
Eif5b	0	0.54721446	0.000616949
Hat1	0	0.546017312	0.032750761
Mut	0	0.545372012	0.044876683
Gm1123	0	0.545239309	0.009368624
Etfa	0	0.54467891	0.011208801
Clca4b	0	0.542832098	0.004044313
Pccb	0	0.541189936	0.028846577

Sptlc2	0	0.541124283	0.020248564
Naip1	0	0.539483153	0.049946816
Mycbp	0	0.539449052	0.035953368
Arhgap5	0	0.538681206	0.008195088
Ankrd49	0	0.536073333	0.043700545
Aldh9a1	0	0.536016085	0.01171753
Rpl4	0	0.535341294	0.0042246
Hsd17b11	0	0.53327119	0.049305256
Apob	0	0.533033046	0.000155406
37500	0	0.532724836	0.015679943
Tmx1	0	0.530309558	0.045372544
Dnajc2	0	0.530047491	0.025624435
Prlr	0	0.529031762	0.001593092
Nfe2l2	0	0.527485348	0.002178857
Ppa1	0	0.527189965	0.032750761
Sod2	0	0.52337514	0.047693037
Dnttip2	0	0.520922013	0.018473563
Strap	0	0.518365829	0.016120511
Atp5f1	0	0.518214116	0.019220398
Atad1	0	0.51785191	0.027764553
Dram2	0	0.51759355	0.045164568
Desi2	0	0.517058306	0.016093418
Rab2a	0	0.514126261	0.006629214
Slc25a46	0	0.511373654	0.020108771
Dut	0	0.510997546	0.042785767
Sptssa	0	0.509043944	0.034786995
Arf4	0	0.506412332	0.021177441
Napepld	0	0.497260209	0.033991007
Usp15	0	0.494387505	0.027116194
Fyco1	0	0.492600189	0.011293246
Ceacam1	0	0.488749252	0.000684616
Cpne3	0	0.488723647	0.002746706
Smarca5	0	0.48593325	0.027764553
Ociad2	0	0.481930328	0.003018953

Pdia3	0	0.481474267	0.000763377
37316	0	0.477605076	0.013231376
Mbd2	0	0.475781708	0.013480475
Pik3c2a	0	0.475726781	0.013185693
Rnf128	0	0.470302593	0.025928916
Aoc1	0	0.467757673	0.014357233
Galnt4	0	0.467292553	0.035953368
Atpif1	0	0.462536505	0.015064034
Krcc1	0	0.45959651	0.033294737
Sdhb	0	0.457571452	0.042770258
Rps21	0	0.457373562	0.033593555
Tm4sf5	0	0.457046778	0.0423098
Acaa2	0	0.450180442	0.039561284
Eif5	0	0.450142674	0.007577129
Cnbp	0	0.449530945	0.004044313
Ssr3	0	0.448179923	0.006541899
Adss	0	0.445004332	0.026602774
Ghitm	0	0.444313114	0.021316141
Tmed7	0	0.44323873	0.045824717
Oat	0	0.442437349	0.002658814
Eif3e	0	0.441560027	0.033524966
Mocs2	0	0.441528068	0.027764553
Golph3	0	0.439822438	0.029017808
Ccar1	0	0.436803004	0.008749016
Paip2	0	0.43138798	0.042105176
B2m	0	0.429970867	0.014174267
Wdr1	0	0.427500515	0.044511453
Hnrnpu	0	0.42628624	0.04146396
Aldob	0	0.425944337	0.009368624
Rps20	0	0.425731381	0.018070308
Ppp2ca	0	0.424546487	0.039537516
Sh3glb1	0	0.423124761	0.037866868
Ywhab	0	0.420048398	0.007880404
Slc5a1	0	0.41746537	0.021177441

Mgam	0	0.415834032	0.001813697
Ltn1	0	0.415396428	0.042494986
Kif5b	0	0.412855842	0.02492109
Man1a	0	0.412420256	0.026602774
Ppp2r3a	0	0.411297806	0.045202582
Suz12	0	0.41086178	0.029222847
Lamp2	0	0.410092879	0.017718898
Rac1	0	0.405962397	0.010356742
Gas5	0	0.405585953	0.028294685
Atp5c1	0	0.402968161	0.035487073
Abcb7	0	0.396734283	0.048571087
Mtpn	0	0.396140091	0.020459064
Tm9sf2	0	0.395145931	0.03681279
Hadh	0	0.394688001	0.042122445
Acat1	0	0.393119914	0.028074029
Erbb2ip	0	0.388493043	0.028074029
Hnrnpm	0	0.387706689	0.024786282
Adgrg7	0	0.385667953	0.017718898
Tspan8	0	0.384650812	0.04108603
Atp5a1	0	0.382824894	0.002746706
Dstn	0	0.382329146	0.031708824
Cul3	0	0.38219626	0.038469737
Atp5g3	0	0.37874831	0.021602712
Purb	0	0.377432488	0.016377774
Ugt2b34	0	0.376962141	0.022469366
Slc44a1	0	0.372987803	0.037927486
Slfn9	0	0.368402783	0.027764553
Slc4a4	0	0.364456018	0.033593555
Apc	0	0.35854367	0.015702583
Me2	0	0.357272819	0.037085605
Qser1	0	0.354511416	0.044356106
Cltc	0	0.353302591	0.030644505
Sdha	0	0.350434184	0.01982306
Atp5b	0	0.349469453	0.006407305

Ipo7	0	0.340553366	0.040626833
Gatad2b	0	0.333216068	0.037681333
Smc4	0	0.327929586	0.045164568
Cdh17	0	0.317433143	0.020248564
Mbnl1	0	0.310649542	0.026602774
Anxa4	0	0.305549286	0.032623252
Tax1bp1	0	0.290093291	0.046189783
Tpr	0	0.285971253	0.042785767
Eif3a	0	0.273564103	0.035422979
Ncl	0	0.268306032	0.04146396
9130208D14Rik	0	0.267450687	0.04626163
Slc12a2	0	0.255690321	0.047693037
Gm8979	0	-0.243920294	0.042770258
Zfhx3	0	-0.342637308	0.038192125
Cdk6	0	-0.344776742	0.014976673
Hcfc1	0	-0.348867498	0.034786995
Tcf20	0	-0.355263871	0.03549296
Utrn	0	-0.366044088	0.010347069
Ptbp1	0	-0.393761419	0.031708824
Rnf43	0	-0.399655078	0.038962099
B4galnt2	0	-0.402411968	0.042907876
Stra6l	0	-0.409479589	0.039537516
Rreb1	0	-0.425205735	0.026575468
Myo9a	0	-0.433163898	0.024786282
Malat1	0	-0.437651491	0.000110881
Trrap	0	-0.448189845	0.035240016
Parp1	0	-0.452420224	0.030523415
Adcy6	0	-0.452714952	0.025928916
Axin2	0	-0.454198177	0.010677467
Pdgfa	0	-0.461926792	0.049369227
B930095G15Rik	0	-0.471968388	0.04146396
Zfp827	0	-0.473693232	0.045372544
Spen	0	-0.476539538	0.026602774
B4galnt1	0	-0.48108776	0.032750761

Neat1	0	-0.496674149	0.015182858
Tnrc18	0	-0.49675897	0.039540173
Foxk1	0	-0.505157121	0.009368624
Ephb4	0	-0.505617822	0.014812066
Kcnq1ot1	0	-0.508492192	0.002860964
Ephb2	0	-0.514359952	0.040626833
Mark2	0	-0.537831854	0.024534138
Prrc2a	0	-0.542788447	0.001229894
Foxp4	0	-0.544841275	0.026849681
Wnk2	0	-0.54909004	0.044876683
Tiam1	0	-0.556408901	0.044511453
Igf1r	0	-0.562059433	0.006629214
Rpl18a	0	-0.576739865	0.022469366
Ncor2	0	-0.580498895	0.016156255
Dazap1	0	-0.589830059	0.025322841
Bcl7a	0	-0.599282964	0.042626146
Arhgap39	0	-0.599503228	0.017718898
Kmt2d	0	-0.609594256	0.000843978
Nav1	0	-0.61896262	0.008069973
Cabin1	0	-0.619796372	0.028918015
Sdc4	0	-0.62699817	0.001328939
Maz	0	-0.64235409	0.040898442
Llgl2	0	-0.661235075	0.046424382
Gm23935	0	-0.674286508	0.012151167
Shisa2	0	-0.675810319	0.018279786
Lamc1	0	-0.71104626	0.041994735
Gm37364	0	-0.713108056	0.001593092
Ergic3	0	-0.713114834	0.006629214
2700049A03Rik	0	-0.716296442	0.040313481
Sec14l1	0	-0.726897859	0.038962099
Lars2	0	-0.728931514	0.000134669
Slc7a5	0	-0.7391996	0.035953368
2210407C18Rik	0	-0.740121825	0.015143495
Dhcr24	0	-0.743575083	0.00741607

Gm38235	0	-0.751772428	0.031481217
Gcn1l1	0	-0.754682167	0.00987359
Zbtb39	0	-0.763917031	0.016672265
Irf1	0	-0.774878339	0.026602774
Gm37159	0	-0.802266418	0.045372544
Sorcs2	0	-0.803859057	0.011849132
Egr1	0	-0.808527185	0.014712583
Iffo2	0	-0.814856225	0.012151167
4930427A07Rik	0	-0.822355703	0.014357233
Capn1	0	-0.835799558	0.010356742
Bcl9l	0	-0.8412542	0.008328412
Csnk1g2	0	-0.842726836	0.016674472
Hspg2	0	-0.864674501	0.009368624
Tmem201	0	-0.883691198	0.009581608
Mecomos	0	-0.88867155	0.035487073
C430002N11Rik	0	-0.889269783	0.012738834
Ubald1	0	-0.896952069	0.048571087
Wnk4	0	-0.897294534	0.031708824
Gm15564	0	-0.921187778	0.000821237
Mbd6	0	-0.923651095	0.005809024
4933426K07Rik	0	-0.968956818	0.033593555
Tpcn1	0	-1.057887152	0.014498252
Rn18s-rs5	0	-1.069959706	2.76E-05
Fam222a	0	-1.121824621	0.033991007
Trp53i11	0	-1.168060499	0.011208801
Kif12	0	-1.17096728	0.027764553
Bbs9	0	-1.319776664	0.005809024
Prpf40b	0	-1.374227138	0.033593555
Cux2	0	-1.471951942	0.049946816
Fosb	0	-1.528825827	0.044344278
Cpm	0	-1.551933079	0.001085044
Igsf9b	0	-1.638960588	0.038847149
Amotl1	0	-1.731063242	0.044344278
Gm44423	0	-1.849110672	0.034786995

Mir6236	0	-2.041836626	5.70E-08
Rab6b	0	-2.118913156	0.044078471
Pgr	0	-2.563135615	0.032616227
Nab2	0	-2.566103268	0.037285233
Slc28a2	0	-3.531286616	0.000134669
Rps4l	0	-5.734130955	0.026602774

Supplemental Table 2. Antibodies used for Western Blot analysis

Type	Antibody	Dilution	Antibody Dilution Buffer
Primary	EGR1 (15F7) Rabbit monoclonal antibody (Cell Signaling Technology, cat. no. 4153)	1:250	5% (w/v) Milk
Primary	IRF-1 (D5E4) XP Rabbit monoclonal antibody (Cell Signaling Technology, cat. no. 8478)	1:250	5% (w/v) BSA
Primary	Recombinant Anti-Fos B antibody (abcam, cat. no. ab184938)	1:1000	5% (w/v) BSA
Primary	Mouse β-actin monoclonal antibody (Santa Cruz, cat. no. sc-47778)	1:1000	5% (w/v) Milk
Secondary	Donkey anti-Rabbit IgG (H+L) Cross-Adsorbed Secondary Antibody, HRP (ThermoFisher Scientific, cat. no. SA1-200)	1:5000	5% (w/v) Milk

Secondary	Donkey anti-Mouse IgG (H+L) Highly Cross-Adsorbed Secondary Antibody, HRP (ThermoFisher Scientific, cat. no. A16017)	1:5000	5% (w/v) Milk
