# Supplementary material for: Intestinal stem cell aging signature reveals a reprogramming strategy to enhance regenerative potential
Source: NPJ Regen Med. 2022 Jun 16;7:31. doi: 10.1038/s41536-022-00226-7 (PMC9203768; doi:10.1038/s41536-022-00226-7)
Supplement: Supplementary file 2 — REPORTING SUMMARY [file 41536_2022_226_MOESM2_ESM.pdf]

nature portfolio

Corresponding author(s): DBPR NPJREGENMED-00648R
Helen E. Abud & Jose M. Polo

Last updated by author(s): Jan 10, 2022

Reporting Summary

Nature Portfolio wishes to improve the reproducibility of the work that we publish. This form provides structure for consistency and transparency in reporting. For further information on Nature Portfolio policies, see our Editorial Policies and the Editorial Policy Checklist.

Statistics

For all statistical analyses, confirm that the following items are present in the figure legend, table legend, main text, or Methods section.

n/a	Confirmed	
☐	☒	The exact sample size (n) for each experimental group/condition, given as a discrete number and unit of measurement
☐	☒	A statement on whether measurements were taken from distinct samples or whether the same sample was measured repeatedly
☐	☒	The statistical test(s) used AND whether they are one- or two-sided<br>*Only common tests should be described solely by name; describe more complex techniques in the Methods section.*
☒	☐	A description of all covariates tested
☐	☒	A description of any assumptions or corrections, such as tests of normality and adjustment for multiple comparisons
☐	☒	A full description of the statistical parameters including central tendency (e.g. means) or other basic estimates (e.g. regression coefficient) AND variation (e.g. standard deviation) or associated estimates of uncertainty (e.g. confidence intervals)
☒	☐	For null hypothesis testing, the test statistic (e.g. F, t, r) with confidence intervals, effect sizes, degrees of freedom and P value noted<br>*Give P values as exact values whenever suitable.*
☒	☐	For Bayesian analysis, information on the choice of priors and Markov chain Monte Carlo settings
☒	☐	For hierarchical and complex designs, identification of the appropriate level for tests and full reporting of outcomes
☐	☒	Estimates of effect sizes (e.g. Cohen's d, Pearson's r), indicating how they were calculated

Our web collection on statistics for biologists contains articles on many of the points above.

Software and code

Policy information about availability of computer code

Data collection	For the organoid experiments, the relative amount of cells per well was evaluated using the Presto Blue cell viability assay (Invitrogen). Fluorescence intensity was measured using a BMG Labtech Fluostar Optima plate reader (excitation: 540 nm; emission: 590 nm).<br>Organoid number and area was analysed using the ImageXpress Pico automated cell imaging system (4x magnification).<br>Imaging of immunohistochemically-stained intestinal tissues was carried out using a Zeiss Axioimager microscope running Axio Vision Rel. 4.7 Software.<br>Sample collection and processing for sequencing experiments (transcriptional and DNA methylation analyses) were performed as described in great detail in the methods section.
Data analysis	Organoid number and area was quantified using the CellReporterXpress image acquisition and analysis software (version 2.8.2.669). The inbuilt Cell Count module was used with the following settings: Intensity - 125, Minimum Width - 20, Maximum Width - 1000.<br>The quantification of DAB signal intensity for EGR1, FOSB and IRF1 was performed using the software Fiji. Following color deconvolution, the average gray value within the crypt domains of DAB-stained pictures was calculated. The optical density per picture was determined as follow: OD = log(255/average gray value).<br>Analysis of sequencing data was performed using publicly available software as per the methods section. Morify predictions based on the transcriptional data were performed as previously described (PMID: 26780608).

For manuscripts utilizing custom algorithms or software that are central to the research but not yet described in published literature, software must be made available to editors and reviewers. We strongly encourage code deposition in a community repository (e.g. GitHub). See the Nature Portfolio guidelines for submitting code & software for further information.

Data

Policy information about availability of data

All manuscripts must include a data availability statement. This statement should provide the following information, where applicable:

- Accession codes, unique identifiers, or web links for publicly available datasets
- A description of any restrictions on data availability
- For clinical datasets or third party data, please ensure that the statement adheres to our policy

Sequencing data have been deposited in the Gene Expression Omnibus (GEO) database with the following accession code GSE198139.

Field-specific reporting

Please select the one below that is the best fit for your research. If you are not sure, read the appropriate sections before making your selection.

☒ Life sciences ☐ Behavioural & social sciences ☐ Ecological, evolutionary & environmental sciences

For a reference copy of the document with all sections, see nature.com/documents/nr-reporting-summary-flat.pdf

Life sciences study design

All studies must disclose on these points even when the disclosure is negative.

Sample size	Sample sizes were determined based on prior experience and published reports on aging in the mouse gut.
Data exclusions	No data were excluded from the analysis.
Replication	Replication was performed at least three times (expect for whole genome bisulfite sequencing, n=1 and single cell RNA sequencing, n=2). Experiments were carried out in biological replicates as indicated.
Randomization	Animals were randomly assigned to different experiments.
Blinding	The investigators were not blinded to group allocation during data collection given the two groups of mice (young and old) demonstrate obvious morphological changes (size and weight).

Reporting for specific materials, systems and methods

We require information from authors about some types of materials, experimental systems and methods used in many studies. Here, indicate whether each material, system or method listed is relevant to your study. If you are not sure if a list item applies to your research, read the appropriate section before selecting a response.

Materials & experimental systems

n/a	Involved in the study	
☐	☒	Antibodies
☒	☐	Eukaryotic cell lines
☒	☐	Palaeontology and archaeology
☐	☒	Animals and other organisms
☒	☐	Human research participants
☒	☐	Clinical data
☒	☐	Dual use research of concern

Methods

n/a	Involved in the study	
☒	☐	ChIP-seq
☐	☒	Flow cytometry
☒	☐	MRI-based neuroimaging

Antibodies

Antibodies used	Antibodies used for immunohistochemistry:<br>anti-EGR1 antibody (15F7), Cell Signaling Technology, #4153; anti-FOSB antibody (5G4), Cell Signaling Technology, #2251; anti-IRF1 antibody (EPR18301), Abcam, #ab186384.<br>Antibodies used for Western-blotting:<br>Anti-EGR1 antibody (15F7), Cell Signaling Technology, #4153; anti-FOSB antibody (EPR15905), Abcam, #ab184938; anti-IRF1 antibody (D5E4), Cell Signaling Technology, #8478; anti-B-ACTIN antibody (C4), Santa Cruz, #sc-47778.<br>The list of antibodies used for cell sorting was provided in detail in our previously published paper (Nefzger et al., A Versatile Strategy for Isolating a Highly Enriched Population of Intestinal Stem Cells, 2016, Stem Cell Reports, volume 6, issue 3, P321-329)
Validation	Antibodies used for immunohistochemistry:<br>Anti-EGR1 antibody (15F7), Cell Signaling Technology, #4153; anti-FOSB antibody (5G4), Cell Signaling Technology, #2251; anti-IRF1

antibody (EPR18301), Abcam, #ab186384. According to manufacturers' websites, these antibodies were validated for immunofluorescence and immunohistochemistry on mouse tissues.
Antibodies used for Western-blotting:
anti-EGR1 antibody (15F7), Cell Signaling Technology, #4153; anti-FOSB antibody (EPR15905), Abcam, #ab184938; anti-IRF1 antibody (D5E4), Cell Signaling Technology, #8478; anti-B-ACTIN antibody (C4), Santa Cruz, #sc-47778. According to manufacturers' websites, these antibodies were validated for western blotting on mouse tissues.

Animals and other organisms

Policy information about studies involving animals; ARRIVE guidelines recommended for reporting animal research

Laboratory animals	2 month-old and 22 month-old wild type C57/Bl6 and Lgr5-EGFP-IRES-CreERT2 female mice were used.
Wild animals	*Provide details on animals observed in or captured in the field; report species, sex and age where possible. Describe how animals were caught and transported and what happened to captive animals after the study (if killed, explain why and describe method; if released, say where and when) OR state that the study did not involve wild animals.*
Field-collected samples	*For laboratory work with field-collected samples, describe all relevant parameters such as housing, maintenance, temperature, photoperiod and end-of-experiment protocol OR state that the study did not involve samples collected from the field.*
Ethics oversight	Experimental procedures were approved by the Monash Animal Research Platform Animal Ethics Committee.

Note that full information on the approval of the study protocol must also be provided in the manuscript.

Flow Cytometry

Plots

Confirm that:

- ☐ The axis labels state the marker and fluorochrome used (e.g. CD4-FITC).
- ☐ The axis scales are clearly visible. Include numbers along axes only for bottom left plot of group (a 'group' is an analysis of identical markers).
- ☐ All plots are contour plots with outliers or pseudocolor plots.
- ☐ A numerical value for number of cells or percentage (with statistics) is provided.

Methodology

Sample preparation	The isolated small intestine was flushed with PBS and opened longitudinally. The tissue was then scraped with a glass coverslip to remove villi, cut into 5-mm pieces and washed with PBS five times (20 inversions of the tube per wash). Following incubation for 30 min at 4°C in 4mM EDTA-PBS, intestinal crypts were released from small intestinal tissue fragments by mechanically pipetting with a 10ml pipette in PBS and repeating this step twice. After centrifugation (1500 rpm for 5 min at 4°C), the pellet was resuspended in PBS and strained using a 70-µm cell strainer (BD Biosciences). Following centrifugation (1500 rpm for 5 min at 4°C), the collected crypts were incubated for 30 minutes at 4°C in DMEM/F12 – 10% serum (Gibco), pelleted again by centrifugation (1500 rpm for 5 min at 4°C) and then dissociated in TrypLE Express (Invitrogen) supplemented with 10 µM Rock inhibitor (Y-27632, Abcam) and 2.5µg/ml DNAse 1 (Sigma-Aldrich) for 4 minutes at 37°C. The dissociated cells were strained using a 70-µm cell strainer and were washed twice with PBS and collected by centrifugation at 4°C at 1500 rpm for 5 minutes. Cellularized intestinal epithelial were labelled with antibodies and ISCs isolated by FACS (SM6 strategy, CD31-/CD45-/CD24med/CD166+/CD44high/GRP78low/EphB2high/Epcam+) as described previously in great detail (Nefzger et al, A Versatile Strategy for Isolating a Highly Enriched Population of Intestinal Stem Cells, 2016, Stem Cell Reports, volume 6, issue 3, P321-329).
Instrument	Cell sorting was carried out on an Influx instrument (BD Biosciences).
Software	Data analysis was performed with the FlowJo Software suite
Cell population abundance	Post sort analysis was routinely performed with >85% of purified life cells falling into the final sort gate.
Gating strategy	Aggregates, debris, dead cells (PI+) and CD45+/CD31+ hematopoietic/endothelial contaminates were depleted. For Lgr5-GFPhigh cells, 2.5-3% of the Lgr5-GFP brightest cells were selected.<br>For the SM6 strategy, the CD24med/CD166low population was subgated into CD44high/GRP78neg-low cells (the gate was set to encompass ~ 33% of the population). An additional step was included where ~33% of the top EPCAMhigh/EPHB2high cells were collected. The gating strategy is described in detail in our method paper: Nefzger et al, A Versatile Strategy for Isolating a Highly Enriched Population of Intestinal Stem Cells, 2016, Stem Cell Reports, volume 6, issue 3, P321-329.

- ☐ Tick this box to confirm that a figure exemplifying the gating strategy is provided in the Supplementary Information.
